# Dynamics of Rye Chromosome 1R Regions with High or Low Crossover Frequency in Homology Search and Synapsis Development

**DOI:** 10.1371/journal.pone.0036385

**Published:** 2012-04-30

**Authors:** Nohelia T. Valenzuela, Esther Perera, Tomás Naranjo

**Affiliations:** 1 Departamento de Genética, Facultad de Biología, Universidad Complutense, Madrid, Spain; 2 Departamento de Biología Vegetal, Facultad de Biología, Universidad Complutense, Madrid, Spain; Université de Sherbrooke, Canada

## Abstract

In many organisms, homologous pairing and synapsis depend on the meiotic recombination machinery that repairs double-strand DNA breaks (DSBs) produced at the onset of meiosis. The culmination of recombination via crossover gives rise to chiasmata, which locate distally in many plant species such as rye, *Secale cereale*. Although, synapsis initiates close to the chromosome ends, a direct effect of regions with high crossover frequency on partner identification and synapsis initiation has not been demonstrated. Here, we analyze the dynamics of distal and proximal regions of a rye chromosome introgressed into wheat to define their role on meiotic homology search and synapsis. We have used lines with a pair of two-armed chromosome 1R of rye, or a pair of telocentrics of its long arm (1RL), which were homozygous for the standard 1RL structure, homozygous for an inversion of 1RL that changes chiasma location from distal to proximal, or heterozygous for the inversion. Physical mapping of recombination produced in the ditelocentric heterozygote (1RL/1RL_inv_) showed that 70% of crossovers in the arm were confined to a terminal segment representing 10% of the 1RL length. The dynamics of the arms 1RL and 1RL_inv_ during zygotene demonstrates that crossover-rich regions are more active in recognizing the homologous partner and developing synapsis than crossover-poor regions. When the crossover-rich regions are positioned in the vicinity of chromosome ends, their association is facilitated by telomere clustering; when they are positioned centrally in one of the two-armed chromosomes and distally in the homolog, their association is probably derived from chromosome elongation. On the other hand, chromosome movements that disassemble the bouquet may facilitate chromosome pairing correction by dissolution of improper chromosome associations. Taken together, these data support that repair of DSBs via crossover is essential in both the search of the homologous partner and consolidation of homologous synapsis.

## Introduction

Chiasma formation between homologous chromosomes at prophase I of meiosis is indispensable for proper reduction of the chromosome number at anaphase I and, hence, for the efficient production of gametes. Chiasmata are formed after culmination of three major processes initiated in early prophase I, homologous pairing (i.e., an interaction of chromosomes that results in the alignment of homologues), synapsis (i.e., the formation of a proteinaceus synaptonemal complex structure between each homologous pair), and crossing over. A crossover and a non-crossover (non-reciprocal exchange) represent the two possible outcomes in the pathway that the homologous recombination machinery follows to repair one DSB generated by the topoisomerase II related enzyme SPO11 at the initiation of meiosis. The majority of DSBs are destined to become non-crossover products; the few that are crossovers create chiasmata forming mechanical bonds between homologues. Crossover and non-crossover pathways diverge at the leptotene-zygotene transition, prior to the formation of extensive strand-exchange intermediates [1].

At the onset of meiosis, homologous chromosomes occupy in many species separate territories [2]. To become paired, they need to be brought into sufficient physical proximity to make feasible the interactions that lead to homology recognition and the establishment of some form of bonds. In the course of leptotene, in most organisms studied, telomeres attach to the inner nuclear envelope and cluster to form the so-called bouquet. Because of the coincidence of synapsis initiation and telomere clustering, the bouquet configuration is regarded as facilitating pairing and synapsis of homologous chromosomes by bringing their telomeres into a close proximity [3]–[9]. Complex networks of interactions between chromosome pairing, synapsis and recombination have been reported [10]. The chromosome homology search, pairing and synapsis are largely dependent on the initiation and progression of recombination in fungi, mammals and plants. However, it is not well understood whether the crossover and non-crossover pathways play a similar role or not in homologous pairing and synapsis.

Meiotic recombination events are non-randomly distributed in the genome, largely because DSBs are more likely to form in some genome regions than in others [11]–[12]. Regions where DSBs occur at relatively high frequency are called recombination hot-spots. A high-resolution map of meiotic DSBs across the genome has been constructed only in two eukaryotes, *Saccharomyces cerevisiae* [13] and mice [14]. In both species, the DSB map displays reasonable agreement with the crossover distribution map. In plants, studies on the distribution of crossovers [15] have been based either on genetic maps or on cytological approaches providing the physical localization of chiasmata or late recombination nodules. These studies have shown that in many plant species with large genomes, such as maize, wheat, barley or rye, the crossover frequency increases with the relative distances from the centromere [16]–[20]. Chiasmata are concentrated in the distal part of most chromosomes in these species. Likewise, chromosome pairing and assembly of the synaptonemal complex are initiated usually at distal sites and succeeded by numerous intercalary initiations [21]–[23].

Chromosome rearrangements such as deletions or inversions, that change the position of chromosome segments on the telomere-centromere axis, are a useful tool in unraveling the role of different chromosome regions in homologous pairing, synapsis and recombination. Heterozygosity for the loss of long terminal segments in wheat chromosomes causes a strong reduction in chiasma frequency in the affected chromosome arm. However, homozygosity for the deletion returns to normal the amount of chiasmata formed [24]–[28]. This behavior of truncated wheat chromosomes suggested that any chromosome region was capable of forming chiasmata when positioned close to the chromosome end. However, a different conclusion was reached from a truncated rye chromosome in a wheat background. The strong reduction of chiasmata caused by a deletion covering the distal 70% of the long arm of rye chromosome 5R (5RL) in both homozygotes and heterozygotes demonstrated that chiasma frequency is region-specific [29]. In spite of the few chiasmata formed by del5RL, synapsis in homozygotes was normal, suggesting different implications of the region-specific DNA sequences in crossover and synapsis.

That it is not the position but the DNA sequence, or chromatin organization, normally present in the distal part of a given chromosome arm that determines the crossover formation, was also demonstrated for the long arm of chromosome 1R (1RL). An inversion covering 90–95% of the 1RL arm in a wheat background was accompanied by a parallel change in the pattern of chiasma distribution [20]. Inversion homozygotes and heterozygotes produced only proximal chiasmata in the inverted arm. However, the behavior of normal and inverted chromosomes 1RL in early meiotic stages was not reported.

The inversion of 1RL represents an excellent chromosome construct to study the role that distal crossover-rich and proximal crossover-poor regions play on homologous partner identification and the initiation and development of synapsis. In contrast with deletions, no chromosome region is lost in the inverted chromosome, which permits to analyze the behavior of different segments in the same cells. In addition, crossover-rich and crossover-poor regions adopt a balanced positioning in heterozygotes; they are located distally in one chromosome and proximally in the homolog, hence avoiding a possible effect of the proximity to the telomere. Rye chromosomes introgressed into wheat can be visualized by fluorescence *in situ* hybridization (FISH) with rye genomic DNA probes or with pUCM600, a rye specific DNA clone [30]. Furthermore, the distal and subdistal C-heterochromatin bands that chromosome 1R usually carries, as well as the centromere, can also be visualized by FISH [29]. In this article, we examine the role that distal crossover-rich and proximal crossover-poor regions of 1RL play in the search of the homologous partner and synapsis through modifications, that the inversion of this arm, caused in the dynamics of such regions in early and mid prophase I. We report on the physical location of crossovers in a heterozygote for the inversion. A majority of crossovers in the arm are formed in a very small region that in a normal chromosome is flanked by a subdistal chromomere and the telomere. In the inverted arm, this region is flanked by a proximal chromomere and the centromere. We conclude that, regardless of their position on the telomere-centromere axis, the chromosome regions with high crossover frequency appear to provide more opportunity for homologous encounters and synapsis than those with low crossover capabilities.

## Results

### Rye chromosome markers

**Figure 1:**
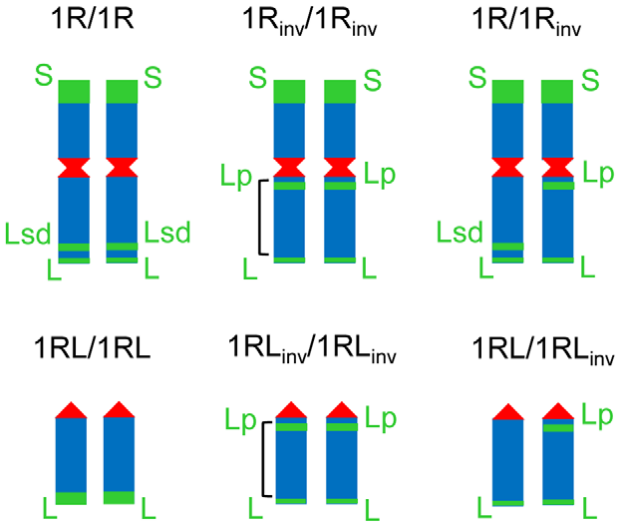
The structure of the rye chromosome pair studied in a wheat background. Disomic introgressed wheat-rye lines for both chromosome 1R and the telocentric of its long arm (1RL) were homozygous for the standard structure (1R/1R and 1RL/1RL) homozygous for a pericentric inversion of its long (1R_inv_/1R_inv_ and 1RL_inv_/1RL_inv_) or heterozygotes (1R/1R_inv_ and 1RL/1RL_inv_). The approximated size of the inversion is indicated in homozygotes. Centromeres (red) and C-heterochromatin blocks S, Lp, Lsd and L (green) are rye-specific chromosome markers identified by FISH.

The structure of mitotic rye chromosomes in each of the six wheat-rye introgressed lines studied is presented in Figure 1. Green bands represent C-heterochromatin chromomeres, which were detected by FISH using clone pSc74. The centromere was detected with clone pAWRC.1 while clone pUCM600 was used to label the remaining rye chromosome regions. The short arm of chromosome 1R carries the largest heterochromatic chromomere (S), and the long arm two smaller chromomeres that are located distally (L) and subdistally (Lsd). Differences in the hybridization signal size identified the small distal and large subdistal chromomere. In the inverted chromosome the subdistal chromomere relocates to the proximity of the centromere (Lp). The ditelocentric heterozygote (1RL/1RL_inv_) lacks the subdistal signal in the 1RL chromosome, which indicates loss of the subdistal chromomere; the standard ditelocentric line (1RL/1RL) carries only a distal large-sized chromomere.

### The position of crossovers in the 1RL arm

**Figure 2:**
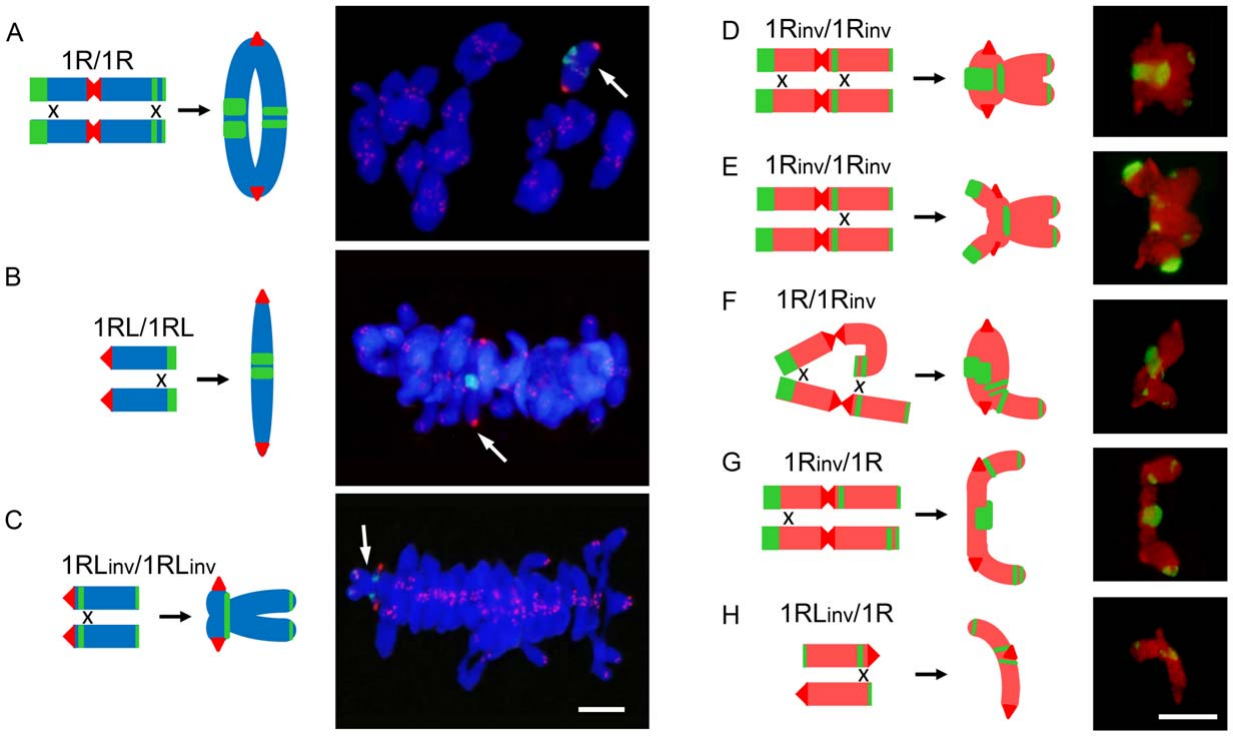
Proximal and distal chiasma location between bi-armed or telocentric rye chromosomes and morphology of bivalents formed at metaphase I in different wheat-rye introgressed lines. Drawings show the position of chiasmata in each bivalent. **A–C**) The rye bivalent (arrow) was identified by FISH with the rye centromere DNA probe pAWRC.1 (strong red signals) and probe pSc74 (green). Telomeres (weak red signals) of all chromosomes were also labeled. Both wheat and rye chromosomes were stained with DAPI. **D–H**) Rye bivalents identified with rye specific DNA probes pUCM600 (red) pAWRC.1 (bright red) and pSc74 (green). Bars represent 10 µm.

At metaphase I (MI) rye chromosomes were paired into bivalents in most pollen mother cells (PMCs) (Fig. 2). Some PMCs with two rye univalents were also observed in inversion homozygotes and heterozygotes and in the normal ditelocentric (1RL/1RL). The frequencies of association of each chromosome arm are given in Table 1. The highest frequencies correspond to lines with the standard chromosome 1R conformation. The inversion caused a considerable reduction in the frequency of bonds in the long arm and changed their position in the chromosome. In normal homozygotes (1R/1R and 1RL/1RL), all bonds between the 1RL arms were distal or subdistal (Fig. 2A, B), they were proximal in inversion homozygotes (1R_inv_/1R_inv_ and 1RL_inv_/1RL_inv_) (Fig. 2C–E), and distal/proximal in the heterozygotes (1R/1R_inv_ and 1RL/1RL_inv_) (Fig. 2F, H). However, chromatin condensation made it difficult to determine the number of chiasmata formed in each bivalent and their exact physical positions.

**Table 1:** Frequency (%) of association of rye chromosome arms 1RL and 1RS at metaphase I in the six wheat-rye lines studied and frequency (%) of bridge+acentric fragment configuration at anaphase I in heterozygotes for the inversion.

Line	Metaphase I			Anaphase I	
	1RS	1RL	PMCs	Bridge+fragment	PMCs
1R/1R	91.1	98.9	90		
1R_inv_/1R_inv_	66.7	62.1	66		
1R/1R_inv_	82.8	20.0	320	20.6	320
1RL/1RL		95.1	102		
1RL_inv_/1RL_inv_		78.8	137		
1RL/1RL_inv_		53.4	318	47.3	317

**Figure 3:**
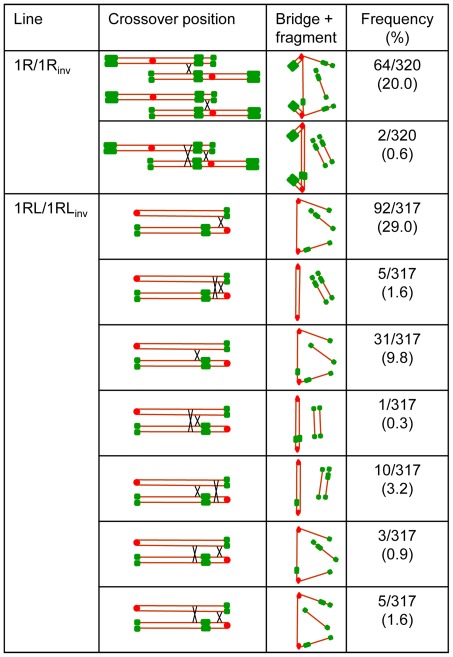
Position and frequency of crossovers that originated each type of bridge and fragment configuration observed at anaphase I in heterozygotes 1R/1R_inv_ and 1RL/1RL_inv_.

**Figure 4:**
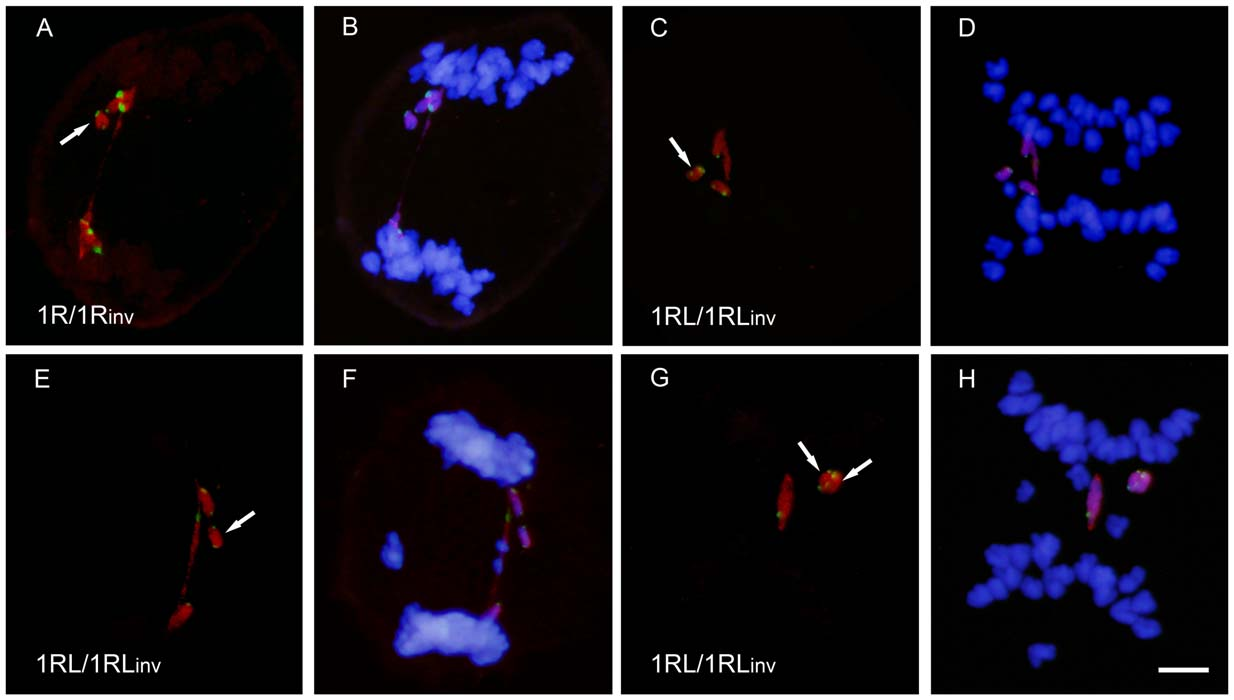
Bridge+fragment configurations formed by rye chromosomes at anaphase I in heterozygotes 1R/1R_inv_ and 1RL/1RL_inv_. Rye chromosomes were identified with DNA probes pUCM600 (red) pAWRC.1 (red) and pSc74 (green) (**A, C, E, G**). Wheat chromosomes were stained with DAPI (**B, D, F, H**). **A,B**) Bridge and fragment formed after one crossover between the long arms of 1R and 1R_inv_. **C–D**) Bridge and three green signals fragment formed by telos 1RL and 1RL_inv_ after one crossover flanked by the centromere and the proximal heterochromatic chromomere of 1RL_inv_. **E, F**) Bridge and two green signals fragment formed after one crossover flanked by the proximal and distal chromomeres of 1RL_inv_. **G, H**) Two bridges and two fragments after two complementary crossovers located at both sides of the proximal chromomere of 1RL_inv_. Bar represents 10 µm.

Chiasma frequency can be estimated in heterozygotes for paracentric inversions (inversions like that of 1RL, which do not include the centromere) from anaphase I (AI) observations. When a single crossover takes place in the inverted segment in such heterozygotes, a bridge+acentric fragment configuration appears at anaphase I (AI) (Fig. 3, Fig. 4). The frequency of bridge+acentric fragment involving the 1RL arm in two-armed and ditelocentric heterozygotes (1R/1R_inv_ and 1RL/1RL_inv_) appears in Table 1. Close correspondence between the frequency of association at MI and the frequency of recombination, as detected by the bridge+acentric fragment configuration at AI (χ2 = 0.04, freedom degrees = 1, p>0.80, for 1R/1R_inv_ and χ2 = 1.17, freedom degrees = 1, p>0.50, for 1RL/1RL_inv_) indicates that bonds at MI were due to chiasmata.

The bridge+acentric fragment configuration provided no information on the crossover situation in the two-armed heterozygote (1R/1Rinv). The numbers and positions of chromomeres in such structures were the same, regardless of the crossover position (Fig. 3, Fig. 4A, B). The infrequent AI PMCs showing two bridges and two fragments indicated a very low frequency of complementary (four chromatid) double crossovers in the two-armed heterozygote (1R/1R_inv_). However, heterozygosity for the subdistal marker in the ditelocentric inversion heterozygote (1RL/1RL_inv_) offered a chance to identify the crossover site, either between the centromere and the proximal chromomere of the inverted chromosome or outside this segment. The number and position of chromomeres in the bridges and acentric fragments at AI change with the crossover position (Fig. 3). A crossover between the centromere and the chromomere results in a three-signal fragment (Fig. 4C, D) while a crossover outside this segment gives rise to a fragment with two signals (Fig. 4E, F). The fragment size equals the 1RL arm length regardless of the crossover position. Two complementary crossovers produced in the same side of the proximal chromomere generate two bridges and two acentric fragments with the labeling patterns as indicated above (Fig. 4G, H). Disparate (three chromatid) double crossover located in the same side of the chromomere could not be identified as they produce the same result at AI as a single crossover. Reciprocal (two chromatid) double crossovers could not be detected as they produce no bridge+fragment configuration at AI. Complementary and disparate double crossovers situated at both sides of the chromomere, which render a diagnostic bridge+fragment labeling, were also detected (Fig. 3).

The crossover frequency in the segment between the centromere and the proximal chromomere was 36.3% and 15.8% in the remainder of the chromosome. This means that 70% all crossovers formed in the arm are located in the very short chromosome segment flanked by the centromere and the proximal chromomere of the inverted chromosome, which has its counterpart in the distal region of the standard chromosome. This segment represents 10% of the pachytene 1RL arm length. The remaining crossovers most likely occur in the immediate vicinity of this segment, as deduced from the proximal position of bonds at MI.

### Dynamics of the rye centromere and distal marker at early and mid prophase I

**Figure 5:**
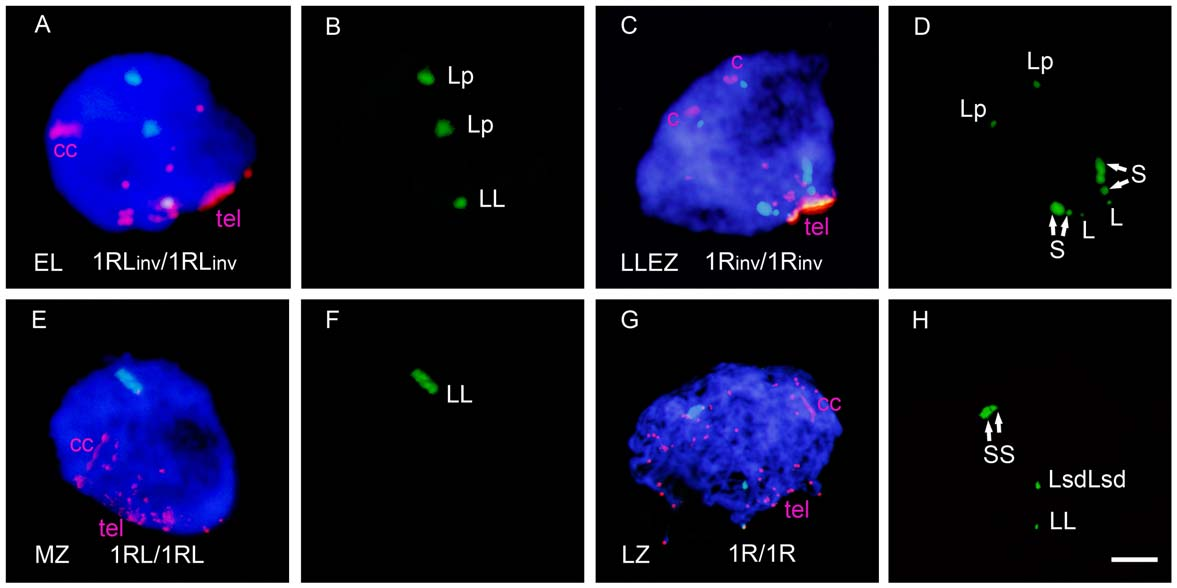
Arrangement of telomeres (tel), rye centromeres (c), and rye heterochromatic chromomeres at early and mid prophase I stages in different rye chromosome combinations. Distal chromomeres of 1RS and 1RL are named S and L, respectively, Lsd designates the subdistal chromomere of the 1RL arm, and Lp the proximal chromomere of 1RL_inv_. **A–B**) Cell at early leptotene (EL) with several telomere groups showing association of the centromeres and distal chromomeres of 1RL_inv_, and separation of the proximal chromomeres. Centromere signals are larger than any telomere signals located in the opposite hemisphere. **C–D**) Cell at the leptotene-zygotene transition (LLEZ) showing a bipolar arrangement of the rye centromeres and the telomere cluster that denotes the bouquet formation. Both centromeres and heterochromatic chromomeres are separated. The S marker appears divided in two unequal subchromomeres (arrows) owing to chromosome elongation. **E–F**) Cell at mid zygotene (MZ) with the bouquet partially disorganized. Centromeres and distal chromomeres are associated. **G–H**) Cell at late zygotene (LZ) with bouquet dissolution. The 1RS subchromomeres (arrows) are joined because of chromatin condensation; all markers are associated. Bar represents 10 µm.

To establish the role that distal and proximal chromosome arm regions play in homology recognition, chromosome pairing and synapsis, the dynamics of the heterochromatic chromomeres and the centromere of the rye chromosome pair were analyzed in meiocytes in stages from early leptotene to pachytene. Meiocyte staging was inferred from the arrangement of telomeres (Fig. 5, S1). In leptotene, telomeres migrate to form a tight cluster and centromeres appear as compact structures located in the opposite pole of the nucleus. In addition, chromatin undergoes a conformational change that results in chromosome elongation [31] which is apparent in centromere signals at the leptotene-zygotene transition. The telomere bouquet is consolidated at the leptotene-zygotene transition and disintegrates at mid zygotene. Late zygotene and pachytene are postbouquet stages that differ in the degree of chromatin condensation. The change of chromatin conformation produced at leptotene showed that the subtelomeric chromomere of 1RS (Fig. 1) subdivides in two, which condense again in one at late zygotene (Fig. 5D, H).

The analysis of the dynamics of homologous chromomeres and centromeres in the course of prophase I was based on changes in the relative position. Two positional categories were considered: absence of association and close physical association. Homologous markers visualized as two FISH signals located at a physical distance higher than 1 µm were scored as non-associated; markers were scored as associated when only the two were fused into one signal, or two touching signals were observed. Examples of associated and non-associated markers are shown in Figure 5.

The distal marker of the 1RS arm, which is not involved in the inversion, behaved the same in the three types of plants studied. Its frequency of association was relatively low at the early leptotene but it increased with telomere clustering at the leptotene-zygotene transition, and especially with the progression of synapsis during zygotene, and reached frequencies close to 100% at pachytene (Fig. S2).

**Figure 6:**
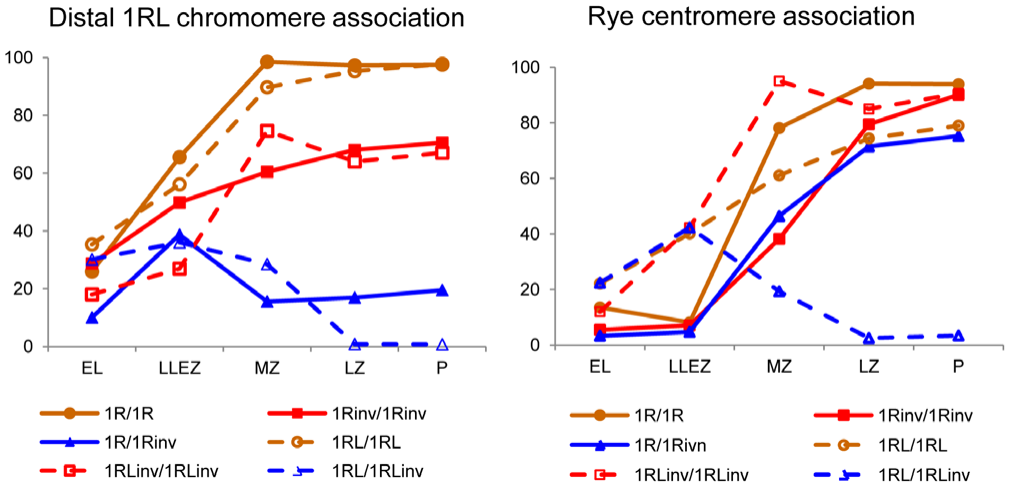
Frequency (%) of association of the distal chromomeres and centromeres of 1RL in early and mid prophase I in the six wheat-rye lines studied. Mean number of PMCs = 159±22.

The behavior of the distal marker on 1RL was affected by the inversion (Fig. 6). In homozygotes for normal arms (1R/1R and 1RL/1RL), the frequency of association of the distal 1RL chromomere, increased throughout the bouquet consolidation and with progression of synapsis, reaching values close to 100% at pachytene. However, inversion homozygotes had the association frequency at pachytene below 70% and even some reduction of the frequency of association after mid zygotene was observed in the ditelocentric homozygote (1RL_inv_/1RL_inv_). This behavior suggests synapsis failure in the distal crossover-poor regions of the 1RL_inv_ chromosome arm. In heterozygotes, the increase in the association frequency of the distal 1RL chromomere, concident with the telomere clustering produced at the leptotene-zygotene transition,was followed of a reduction during the synaptic development that caused almost complete disappearance of the initial associations in the ditelocentric heterozygote (1RL/1RL_inv_).

The behavior of centromeres in all lines also is illustrated in Figure 6. Remarkable is the increase of associations at the leptotene-zygotene transition in all three ditelocentric lines, which is absent in lines with two-armed chromosomes. Centromeres of telocentric chromosomes migrate to the telomere pole most likely dragged along by their telomeres during bouquet formation. Hence, they have more opportunities of bringing together than centromeres of bi-armed chromosomes, which remain at the centromere pole. The levels of centromere associations increased with the progression of synapsis in the ditelocentric homozygotes, but homozygotes for the inversion reached a higher level of association than normal homozygotes. Thus, the centromere behavior changes with the proximity of the crossover-rich region. In contrast with homozygotes, most centromere associations present at the bouquet stage in the ditelocentric heterozygote (1RL/1RL_inv_) were dissolved in zygotene. Centromere and distal marker behave the same in the ditelocentric heterozygote. Among the lines with two-armed chromosomes, the final frequency of association was higher than 90% in the two homozygotes, although associations of the centromeres of inversion chromosomes were delayed relative to that of standard chromosomes, and close to 80% in the heterozygote. The different behaviors of centromeres in the two types of heterozygotes is most likey the result of the presence or absence of a short arm. Association of centromeres of the two-armed chromosomes could be produced as an extension of the short arm synapsis.

### Development of synapsis of the rye chromosome pair

**Figure 7:**
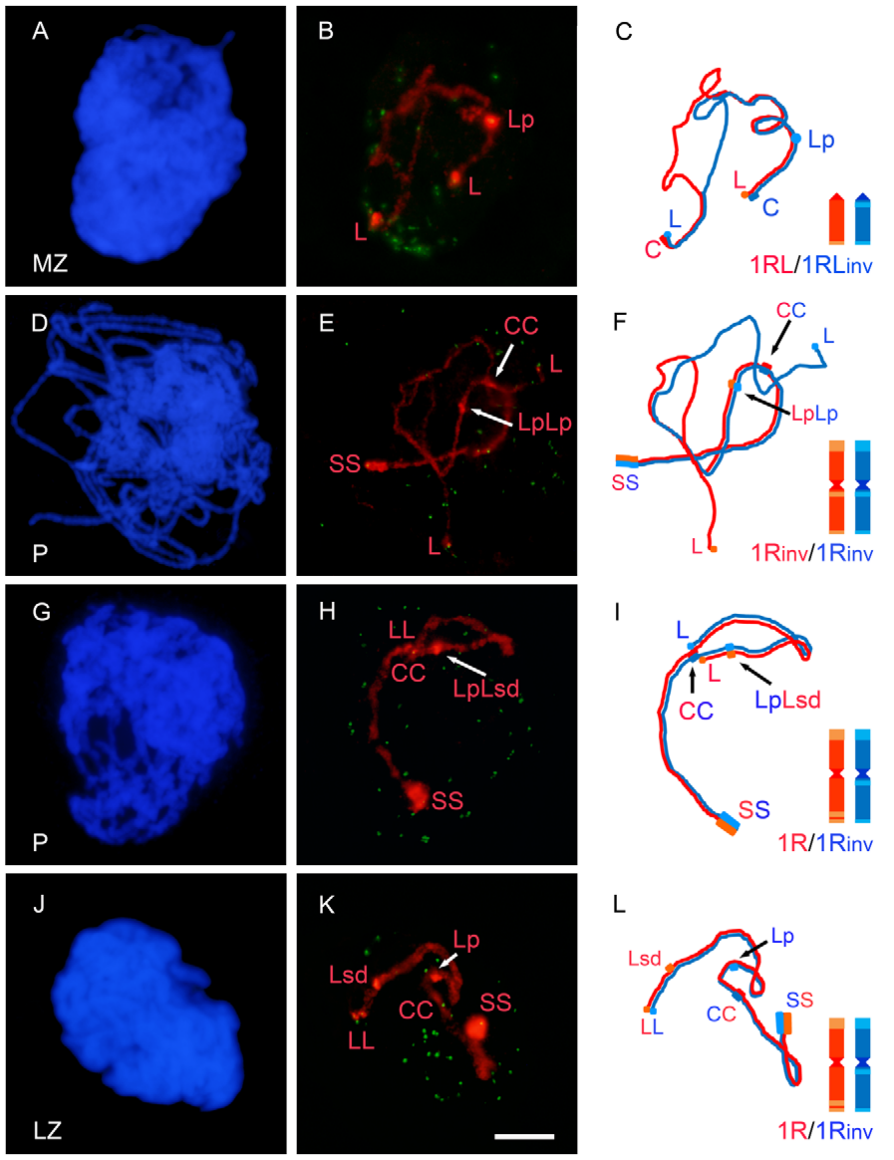
Synaptic configuration of the rye chromosome pair in cells at mid zygotene (MZ), late zygotene (LZ) and pachytene (P) in different lines. **A, D, G, J**) DAPI image of each nucleus. **B, E, H, K**) Arrangement of telomeres labeled with probe pAt74 (green) and of the rye bivalent hybridized with probes, pUCM600, pAWRC.1, pSc74 (red) present in each nucleus. **C, F, I, L**) Schematic representation of the two rye homologues that synapse in each bivalent. **B, C**) 1RL and 1RL_inv_ show antiparallel arrangement and synapsis at both ends. **E, F**) Synapsis of the 1R_inv_-1R_inv_ pair involves 1RS and the proximal region of 1RL_inv_ including the proximal chromomere. **H, I**) Chromosomes 1R and 1R_inv_ show complete homologous synapsis. **K, L**) Chromosomes 1R and 1R_inv_ underwent homologous synapsis of the short arm and non-homologous synapsis of the long arm. Bar represents 10 µm.

To confirm the effect that the inversion of crossover-rich and crossover-poor chromosome regions caused in the synaptic pattern, we quantified the progression of synapsis in meiocytes at zygotene and pachytene. Cells studied were grouped in three classes: PMCs with asynapsis (synapsis level = 0%), PMCs with partial synapsis (synapsis level <90%), and PMCs with complete synapsis (synapsis level >90%). Some examples are shown in Figure 7. Distinction was made between the short and the long arms in the classification of the meiocytes. Only the results of mid zygotene, late zygotene and pachytene are shown as the synapsis level at the leptotene-zygotene transition was low. The 1RS arm completed synapsis in most PMCs at late zygotene in all the three lines, even though the completion was reached somewhat earlier in the standard homozygote than in the other two lines (Fig. S3).

**Figure 8:**
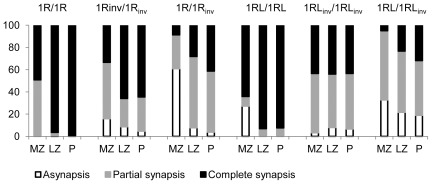
Frequency (%) of PMCs with asynapsis, partial synapsis or complete synapsis of the 1RL arm in the six types of plants studied. Among PMCs of the 1R/1R_inv_ heterozygote with complete synapsis, 45% showed non-homologous synapsis and 55% homologous synapsis. Mean number of PMCs = 119±16.

The level of synapsis of the 1RL arm in all of the lines studied appears in Figure 8. Homozygotes for the standard arm structure (1R/1R and 1RL/1RL) completed synapsis of 1RL at the end of zygotene in most PMCs. Nevertheless, the number of cells with asynapsis at mid zygotene suggests that the initiation of synapsis was delayed in the ditelocentic heterozygote. The frequency of PMCs with complete synapsis decreased in inversion homozygotes. In addition to some degree (5%–7%) of asynapsis, 31% of PMCs at pachytene, in two-armed homozygotes (1R_inv_/1R_inv_), and 50% of PMCs, in ditelocentric homozygotes (1RL_inv_/1RL_inv_), showed partial synapsis. The synaptic pattern indicates that, in the ditelocentric homozygote, progression of synapsis stopped earlier than in the bi-armed homozygote. Heterozygotes showed levels of synapsis lower than homozygotes for the inversion. Only 41% of PMCs at pachytene, in the bi-armed heterozygote (1R/1R_inv_), and 32%, in the ditelocentric (1RL/1RL_inv_), completed synapsis. All of these PMCs developed homologous synapsis in the ditelocentric heterozygote. Among PMCs that completed synapsis at pachytene in the bi-armed heterozygote, only 55% developed homologous synapsis (Fig. 7H, I) while 45% had non-homologous synapsis (Fig. 7K, L).

Matched chromosome segments in PMCs with partial synapsis, in homozygotes for the inversion, concerned either proximal regions or distal regions, or both, but with a different frequency (Table 2). Synapsis failure mainly affected the distal crossover-poor region. In heterozygotes, proximal and distal chromosome segments of 1RL and 1RL_inv_ were found matched in all possible combinations, in PMCs with partial synapsis. These combinations and the number of PMCs scored are also show in Table 2. Proximal regions of 1RL and 1RL_inv_ were often matched at late zygotene and pachytene in the two-armed heterozygote (1R/1R_inv_), probably as an extension of synapsis produced in the short arm since, in most cases, the synapsed stretch covered only the centromere region. Association of the distal regions was rare at these stages. Among the proximal-distal combinations, synapsis between proximal 1RL_inv_ and distal 1RL was much more frequent than synapsis between distal 1RL_inv_ and proximal 1RL. This result suggests that distal 1RL and proximal 1RL_inv_, that is to say, the homologous crossover-rich regions, find each other more easily than other arm combinations.

## Discussion

The study of pairing at MI confirms the change in chiasma location from distal to proximal associated with the inversion of 1RL in homozygotes and heterozygotes [20]. Although this inversion did not affect homology recognition and synapsis of the 1RS arm, pairing of 1RS at MI was reduced in the inversion homozygotes. Such a reduction is line-specific and not affected by the inversion [20]. By contrast, a much higher reduction of metaphase I pairing for the 1RL arm in homozygotes and heterozygotes for the inversion was, in fact, accompanied of failure on homologous recognition and synapsis. The results obtained for the behavior of the centromere and distal marker of 1RL at early meiosis and the synaptic pattern of this arm provide relevant information concerning the dynamics of regions with high and low crossover frequency in the partner identification and initiation and progression of synapsis.

### Does the crossover distribution observed here apply to a standard 1RL arm?

The absence of chiasmata in the proximal halves of chromosomes is common to many triticeae species including wheat and rye. With respect to the physical mapping of crossovers, this study has identified a physically short chromosome region that harbors 70% of all crossovers in the ditelocentric heterozygote (1RL/1RL_inv_). The crossover-rich region is flanked by the centromere, on one side, and the proximal chromomere of the inverted chromosome, on the other. At pachytene, it represents 10% of the 1RL_inv_ arm length. A homologous segment of comparable length located distally in the 1RL arm is flanked by the distal and subdistal chromomeres in the in the standard two-armed homozygote (1R/1R). Rye chromosomes are polymorphic for these cytological markers, which was used to estimate a frequency of recombination of 3.1% for this segment and 48.7% for the adjacent segment between the subdistal chromomere and a third chromomere situated in the middle of the arm [32]. Some methodological difficulties in identification of parental and recombinant chromosome types could, at least in part, explain the low recombination frequency in the most distal segment of 1RL.

The crossover frequencies estimated in the ditelocentric heterozygote (1RL/1RL_inv_), are lower than those of the standard 1RL arm, as deduced from the number of bonds at MI. A high resolution genetic map of rye lists 1RL at 74 cM [33]. An estimate of the genetic length of 1RL can also be obtained from the frequency of PMCs with one and two crossover as follows. The genetic length (L) of a chromosome segment where one crossover occurs with frequency f_1_, two crossover with frequency f_2_, three crossovers with frequency f_3_, and so one, can be calculated as L = 50(f_1_+2f_2_+3f_3_+…nf_n_) [34]. The frequency of PMCs with one and two crossovers in the 1RL/1RL_inv_ heterozygote yields a genetic length of 26.97 cM, or 36% of recombination in the standard 1RL arm. Such a ratio seems to be sufficient to consider the crossovers distribution observed in the inversion heterozygote as representative of the crossover distribution in a standard chromosome arm.

One can argue that wheat chromosomes might affect the number and distribution of crossovers in the 1RL arm. Location of chiasmata in the distal half of the standard 1RL is maintained in the disomic wheat-1R and wheat-1RL introgressed lines. Wheat chromosomes were found to cause a reduction of the number of chiasmata of the 1RL arm only when a large subtelomeric heterochromatin block was present, especially in heterozygous condition. No effect was apparent in the absence of rye subtelomeric heterochromatin [35]. Accordingly, the delay of synapsis initiation detected in the standard ditelocentric (1RL/1RL) could have been generated by the larger size of its distal chromomere. The high degree of chromatin compactation in distal chromomeres may increase resistance to movement during telomere migration and large chromomeres could be expected to move more slowly than small-sized chromomeres.

### Intrachromosomal differentiation of 1RL in the control of homologous pairing, synapsis and recombinatation

Homozygosity and heterozygosity for the inversion were informative with respect to the different roles that the crossover-rich and crossover-poor regions may play in the search for their homologous partners and in the synaptic development. Homozygosity for the inversion produced synapsis failure in the distal region of the inverted arm, that is, in the region normally with a low crossover frequency. The synaptic failure occurred regardless of whether the accompanying subtelomeric markers were associated or not, which means that such a region was much less efficient than the high crossover frequency region in the processes of homologous alignment and assembly of the synaptonemal complex.

Differences between heterozygotes in the synaptic pattern can be explained by the presynaptic arrangement of chromosomes and a different behavior of centromeres of telocentric and bi-armed chromosomes at the bouquet organization. At the onset of meiosis chromosomes retain the geography of the previous anaphase, the Rabl model [31], [36], and the homologous domains of 1RL and 1RL_inv_ occupy territories located in opposite poles of the nucleus, that is, one is positioned close to the telomere, hence in the telomeric pole of the nucleus, while the other is by the centromere, hence in the opposite pole (Fig. S1). The initial spatial separation of the homologous distal 1RL and proximal 1RL_inv_, or proximal 1RL and distal 1RL_inv_, regions disappears with the bouquet organization in the case of the telocentric chromosomes; telomere clustering obliges the centromere of telocentric chromosomes to move to the telomere pole [31], [36]. This chromosome movement facilitates the occurrence of interactions between homologous regions and only homologous synapsis is produced. However, interactions leading to stable synapsis occur more often between the crossover-rich distal 1RL and proximal 1RL_inv_ regions than between the crossover-poor combination.

The situation is completely different in the bi-armed heterozygote (1R/1R_inv_), in which, the antiparallel orientation of 1RL and 1RL_inv_ was not affected by telomere clustering. Although the telomeric or subtelomeric homologous stretches of 1RL and 1RL_inv_ not included in the inversion may interact at the bouquet stage, the progression of synapsis toward the chromosome center is complicated by the absence of homology. Nevertheless, approximately one half of completely synapsed bivalents at pachytene concluded their non-homologous synapsis. Chromosomes that do not find the homologous partner may synapse non-homologously, as it happens in haploid rye [37]. However, homologous regions of the bi-armed chromosomes located in opposite poles of the nucleus were still capable of interactions in cells with homologous synapsis. Such homologous interactions must depend on chromosome movements generated by a mechanism different of telomere clustering. Concomitant with bouquet organization, chromatin undergoes a decondensation process that leads to approximately a five-folds enlargement of the chromosome length [31], [36]. This chromatin remodeling does not increase the nuclear size, hence the elongated chromosomes must move and may span the entire nucleus. These chromosome movements, assumed to occur without any programmed orientation, may generate chance encounters between homologous regions, even if they initially located at very distant spatial territories of the nucleus. Such interactions occur also more often between crossover-rich regions than between crossover-poor regions.

**Table 2:** Matched regions in meiocytes with partial synapsis at mid zygotene (MZ), late zygotene (LZ) and pachytene (P) in homozygotes and heterozygotes for the inversion.

Line	Stage	Matched regions in homozygotes						Total PMCs
		pi-pi	di-di	pi-pi+ di-di				
1R_inv_/1R_inv_	MZ	41	25	20				138
	LZ	41	2	0				126
	P	35	7	0				107
1RL_inv_/1RL_inv_	MZ	28	5	32				122
	LZ	30	8	10				100
	P	36	3	14				106

		Matched regions in heterozygotes						
		pn-pi	dn-di	pn-pi+ dn-di	pn-di	pi-dn	pn-di+ pi-dn	
1R/1R_inv_	MZ	0	5	11	6	10	0	105
	LZ	62^a^	0	5	0	31	0	153
	P	45^a^	0	0	0	28	0	133
1RL/1RL_inv_	MZ	14	31	24	5	25	19	186
	LZ	3	3	2	0	60	1	125
	P	1	5	0	0	66	1	148

p = proximal; d = distal; n = 1RL; i = 1RL_inv_.

a Synapsis covered only the centromere region in 74% of PMCs at MZ and 89% of PMCs at P.

Thus, the distal 10% of the 1RL arm not only harbors a good part of all crossovers produced in this arm, but it is also essential in the search for the homologous partner and the initiation, and development, of synapsis. This implies that chromosome pairing synapsis and crossing over are DNA sequence-dependent. In addition, they are interconnected by the multifunctional activity of recombination proteins Mer3, Msh4 and Mlh1. These proteins that are implicated in recombinational interactions of the crossover pathway during the leptotene/zygotene transition, zygotene and pachytene, play also a direct role in partner identification and chromosome pairing [38]. The existence of a specific region involved in all three processes: homologous pairing, synapsis and crossing over, and of other regions that do not seem to be involved with any of the three, indicates an intrachromosomal differentiation of 1RL in the control of meiotic events. Whether this differentiation is specific to just 1RL or some general feature of cereal chromosomes is an open issue. In two reverse tandem duplications in wheat involving almost complete arms, chiasma locations were restricted to the same exact positions as in structurally normal arms; long regions of arms that in a normal arm are proximal and which were placed by the duplication-inversion at the telomeres, were never involved in chiasma formation, very much like the 1RL here [39]. The long arm of rye chromosome 5R also shows distal or subdistal chiasmata but the role of its proximal part seems to be different than in 1RL; deletion of the crossover-rich distal region of 5RL does not affect pairing and synapsis of the remaining portions of the chromosome [29].

### Bouquet disorganization accompanies pairing and synapsis correction

Our results address not only the dynamics of the presynaptic chromosome movements with respect to the homology search and synaptic development but also the chromosome movements following the initiation of synapsis. Dispersion of the chromosome ends during bouquet dissolution can be counted among intranuclear meiotic movements. Separation of telomeres during zygotene is accompanied by a reduction in the level of association of the distal chromomeres in heterozygotes, while the proximal and distal homologous regions continue to interact. All associations detected at the leptotene-zygotene transition between the terminal chromomeres in the ditelocentric heterozygote, and half of those produced in the bi-armed heterozygote (1R/1R_inv_), did not develop stable synapsis and were lost with the telomere dispersion (Fig. 6). Likewise, most of the centromere associations observed at the bouquet stage in the ditelocentric heterozygote (1RL/1RL_inv_) disappeared during zygotene. Such a behavior suggests that bouquet dissolution facilitates a correction of pairing and synapsis by elimination of improper and unstable chromosome associations, allowing homologues to develop more stable interactions. The difference between stable and unstable associations might depend on the capability that they have to form a crossover or not.

This is likely the pairing correction mechanism that operates in polyploid wheats, where homologous and homoeologous chromosomes compete for pairing at the onset of meiosis, and a considerable number of multivalents can be observed at the early and mid zygotene. Such multivalents formed by homologous and homoeologous chromosomes are reduced to homologous bivalents at late zygotene and pachytene in the wild *Ph1* genotype [21], [40]–[41]. The *Ph1* locus is responsible for the diploid-like behavior of polyploids wheats, which form only bivalents at MI. Chiasmata are formed only between homologous chromosomes in the presence of *Ph1* but, when *Ph1* is absent, chiasmata can also be formed between homoeologues, and multivalents persist until MI [42]–[43]. Restriction of crossovers to homologous chromosomes in the wild type wheat may be responsible of the instability of homoeologous synapsis, which disappears during the bouquet dissolution stage, thus facilitating completion of synapsis between homologous chromosomes. In the absence of *Ph1*, the repair of DSBs via crossover between homoeologues is permitted, stabilizing homoeolgous synapsis in the prophase I multivalents, which therefore can reach MI.

## Materials and Methods

### Plant material

Six wheat-rye introgression lines were used in this study. Three of these lines carried the chromosome pair 1R of rye (*S. cereale*), and the other three had two telocentrics for the 1RL arm, introgressed in the genetic background of hexaploid wheat, *Triticum aestivum*. Each set of three lines consisted of homozygotes for the standard chromosome structure (1R/1R and 1RL/1RL, respectively), homozygotes for the inversion of 1RL (1R_inv_/1R_inv_ and 1RL_inv_/1RL_inv_, respectively), and heterozygotes (1R/1R_inv_ and 1RL/1RL_inv_, respectively). Chromosome stocks 1R/1R, 1R_inv_/1R_inv_, 1RL_inv_/1RL_inv_ and two progenies of the 1RL/1RL_inv_ heterozygote were generated in the genetic background of hexaploid wheat cv. Pavon 76, as substitutions for chromosome 1A, by A.J. Lukaszewski, Univ. of California, Riverside, USA [20], and provided to the authors for further study. The 1RL/1RL_inv_ heterozygote studied was isolated among the above two progenies and the 1R/1RL_inv_ heterozygote was obtained in a cross between homozygotes 1R/1R and 1R_inv_/1R_inv_. The wheat-1RL/1RL homozygote is a ditelocentric line derived from the Chinese Spring-1R addition line [44]. All plants used were grown in a greenhouse from November to May under natural light. At meiosis, one of the three anthers of each flower was checked to establish the meiotic stage and the other two were fixed in 3∶1 ethanol acetic acid, and stored at 4°C.

### Fluorescence in situ hybridization

Fixed anthers were digested in a pectolytic enzyme mixture, transferred to a clean slide and pretreated as previously described [31]. For the study of the arrangement of centromeres and heterochromatic chromomeres of rye chromosomes at prophase I, the following DNA probes were used: clone pAWRC.1 containing a rye-specific centromere repeat [45], clone pSc74 containing a rye-specific 480-bp tandem repeat [46]–[47] and clone pAt74 containing the *Arabidopsis* telomere tandem repeat [48]. For the study of synapsis a fourth DNA probe containing a rye-specific repeat, clone pUCM600 [30], was added. Rye chromosomes at MI were identified with all three rye-specific DNA probes pSc74 and pAWRC.1 and pUCM600, or only with pSc74 and pAWRC.1. Anthers at anaphase I-telophase I of heterozygotes 1R/1R_inv_ and 1RL/1RL_inv_ were analyzed with probes pUCM600, pSc74 and pAWRC.1. Probe concentrations in the different hybridization mixes were 5 ng/µl, for pAt74, and 10 ng/µl, for probes pAWRC.1, pSc74 and pUCM600. Probe pAt74 that labels both wheat and rye telomeres was used in the identification of the prophase I stage as previously described [29], [49].

All clones were labeled by nick translation with biotin-16-dUTP or digoxigenin-11-dUTP. In the analysis of the position of centromeres and chromomeres of rye chromosomes, probes pAt74 and pAWRC.1 were labeled with biotin-16-dUTP and probe pSc74 with digoxigenin-11-dUTP. In the analysis of synapsis, rye-specific DNA probes pAWRC.1, pSc74, and pUCM600 were labeled with biotin-11-dUTP and the telomeric DNA probe pAt74 with digoxigenin-11-dUTP. In the analysis of recombination at anaphase I-telophase I, probes pUCM600 and pAWRC.1 were labeled with biotin-11-dUTP and probe pSc74 with digoxigenin-11-dUTP. The digoxigenin-labelled probes were detected with 6–8 ng/µl of the FITC-conjugated antidigoxigenin antibody (Sigma, St Louis) in 4B (0.5% blocking reagent in 4×SSC) and biotin-labelled probes with 10–15 ng/µl of the Cy3-conjugated avidine (Sigma) in 4B.

Images of cells were viewed under an Olympus BX60 fluorescence microscope equipped with an Olympus DP70 CCD camera. Images were optimized for brightness and color using Adobe Photoshop CS4.

## Supporting Information

Figure S1 **Arrangement of rye chromosomes at early meiosis in two-armed (A) and ditelocentric (B, C) inversion heterozygotes.** A–B) Nuclei at early leptotene (EL) with rye chromosomes (red) positioned in separated territories. The arms 1RL and 1RL_inv_ show antiparallel orientation as it is indicated in the diagrams. Rye chromatin is still higly compacted and telomeres (green) form several miniclusters. C) Nucleus at the leptotene-zigotene transition (LLEZ) with a tight telomere cluster and apparent chromatin decondensation. The centromere of 1RL remains at the centromere pole while the centromere of 1RL migrated to the telomere pole. Bar represents 10 µm.
(TIF)

Figure S2 **Frequency (%) of association of the distal 1RS chromomere pair in early and mid prophase I in plants 1R/1R, 1R_inv_/1R_inv_ and 1R/1R_inv_.** EL, early leptotene; LLEZ, late leptotene-early zygotene; MZ, mid zygotene; LZ, late zygotene; P, pachytene. Mean number of PMCs = 181±32.
(TIF)

Figure S3 **Frequency (%) of PMCs with asynapsis, partial synapsis or complete synapsis of the 1RS arm in plants 1R/1R, 1R_inv_/1R_inv_ and 1R/1R_inv_.** Mean number of PMCs = 126±20.
(TIF)
