# Supplementary figures and images for: Dynamics of Rye Chromosome 1R Regions with High or Low Crossover Frequency in Homology Search and Synapsis Development
Source: PLoS One. 2012 Apr 30;7(4):e36385. doi: 10.1371/journal.pone.0036385 (PMC3340359; doi:10.1371/journal.pone.0036385)

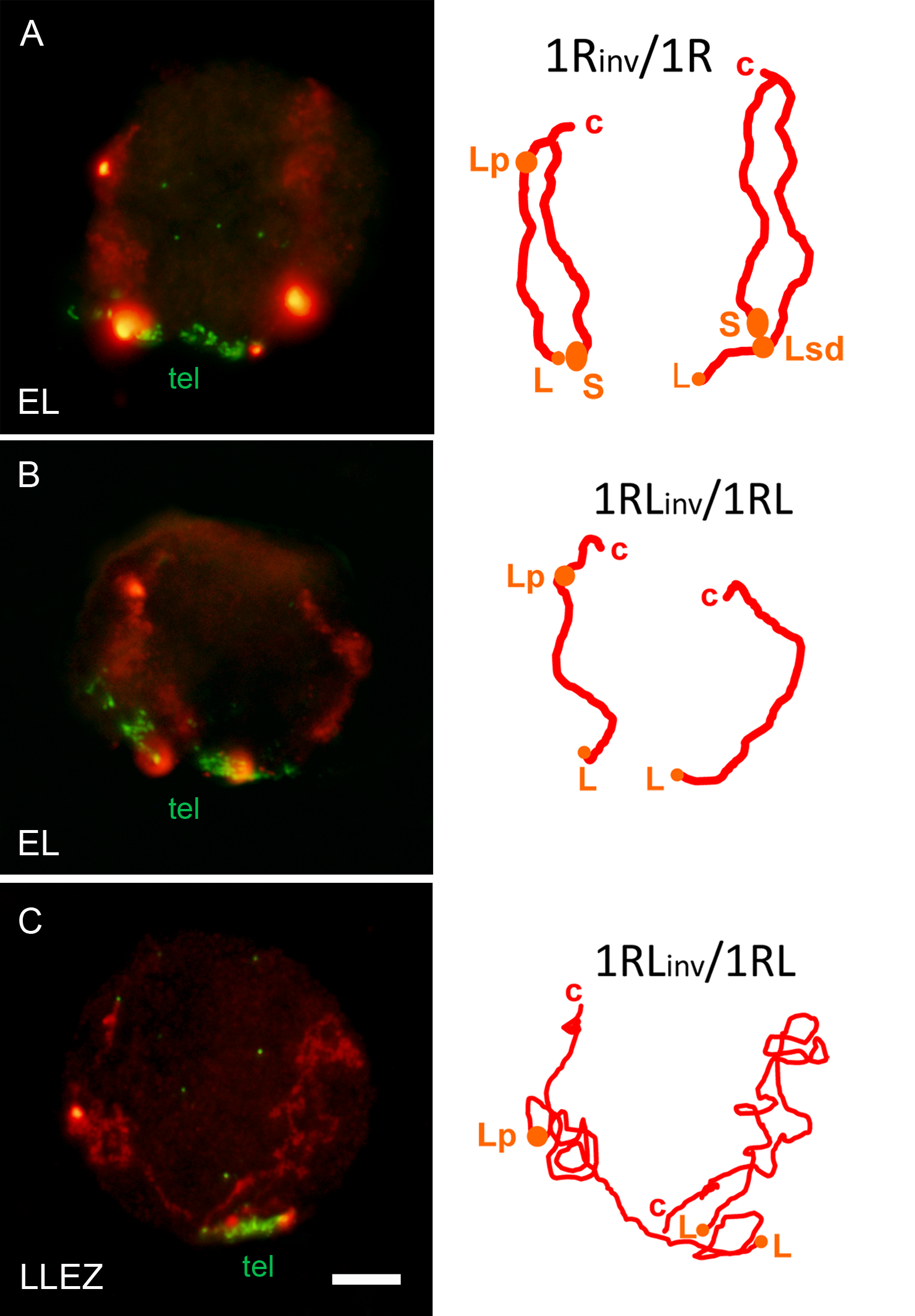

Supplement: Figure S1 — Arrangement of rye chromosomes at early meiosis in two-armed (A) and ditelocentric (B, C) inversion heterozygotes. A–B) Nuclei at early leptotene (EL) with rye chromosomes (red) positioned in separated territories. The arms 1RL and 1RLinv show antiparallel orientation as it is indicated in the diagrams. Rye chromatin is still higly compacted and telomeres (green) form several miniclusters. C) Nucleus at the leptotene-zigotene transition (LLEZ) with a tight telomere cluster and apparent chromatin decondensation. The centromere of 1RL remains at the centromere pole while the centromere of 1RL migrated to the telomere pole. Bar represents 10 µm. (TIF) [file pone.0036385.s001.tif]

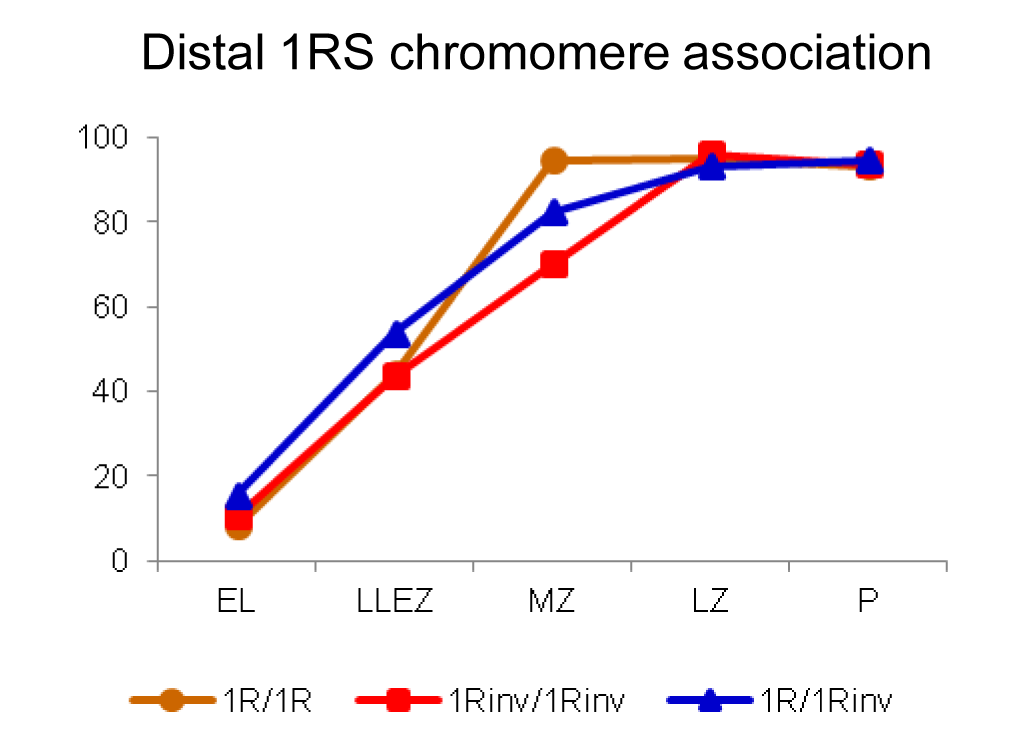

Supplement: Figure S2 — Frequency (%) of association of the distal 1RS chromomere pair in early and mid prophase I in plants 1R/1R, 1Rinv/1Rinv and 1R/1Rinv. EL, early leptotene; LLEZ, late leptotene-early zygotene; MZ, mid zygotene; LZ, late zygotene; P, pachytene. Mean number of PMCs = 181±32. (TIF) [file pone.0036385.s002.tif]

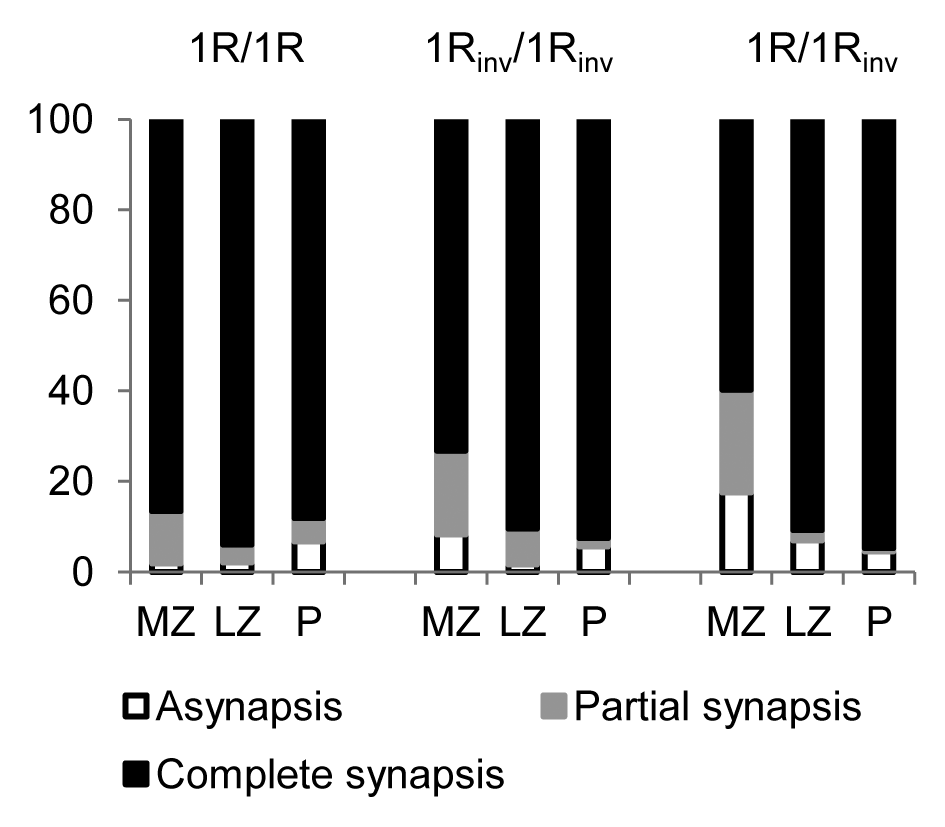

Supplement: Figure S3 — Frequency (%) of PMCs with asynapsis, partial synapsis or complete synapsis of the 1RS arm in plants 1R/1R, 1Rinv/1Rinv and 1R/1Rinv. Mean number of PMCs = 126±20. (TIF) [file pone.0036385.s003.tif]
